# Convolutional neural network deep learning model accurately detects rectal cancer in endoanal ultrasounds

**DOI:** 10.1007/s10151-024-02917-3

**Published:** 2024-04-01

**Authors:** D. Carter, D. Bykhovsky, A. Hasky, I. Mamistvalov, Y. Zimmer, E. Ram, O. Hoffer

**Affiliations:** 1 Department of Gastroenterology, Chaim Sheba Medical Center, Ramat Gan, Israel; 2 Faculty of Medicine, Tel Aviv University, Tel Aviv, Israel; 3 Electrical and Electronics Engineering Department, Shamoon College of Engineering, Beer-Sheba, Israel; 4 School of Electrical Engineering, Afeka College of Engineering, Tel Aviv, Israel; 5 School of Medical Engineering, Afeka College of Engineering, Tel Aviv, Israel

**Keywords:** Rectal cancer, Endoanal ultrasound,deep learning, Convolutional neural network

## Abstract

**Background:**

Imaging is vital for assessing rectal cancer, with endoanal ultrasound (EAUS) being highly accurate in large tertiary medical centers. However, EAUS accuracy drops outside such settings, possibly due to varied examiner experience and fewer examinations. This underscores the need for an AI-based system to enhance accuracy in non-specialized centers. This study aimed to develop and validate deep learning (DL) models to differentiate rectal cancer in standard EAUS images.

**Methods:**

A transfer learning approach with fine-tuned DL architectures was employed, utilizing a dataset of 294 images. The performance of DL models was assessed through a tenfold cross-validation.

**Results:**

The DL diagnostics model exhibited a sensitivity and accuracy of 0.78 each. In the identification phase, the automatic diagnostic platform achieved an area under the curve performance of 0.85 for diagnosing rectal cancer.

**Conclusions:**

This research demonstrates the potential of DL models in enhancing rectal cancer detection during EAUS, especially in settings with lower examiner experience. The achieved sensitivity and accuracy suggest the viability of incorporating AI support for improved diagnostic outcomes in non-specialized medical centers.

## Introduction

Imaging is crucial in evaluating rectal cancer for staging, therapeutic strategy planning, treatment response assessment, and follow-up. The local and distal extent of the disease is typically evaluated with endoanal ultrasound (EAUS) to facilitate the staging of rectal cancer [1]. In an EAUS, the rectal tumor displays as a hypoechoic mass that disrupts the normal echo-layer pattern of the rectal wall [2]. Meta-analysis of EAUS staging data has demonstrated pooled sensitivity estimates of 80.5–96.4% and pooled specificity estimates of 90.6% to 98.3% for cancer detection [3]. Moderate but acceptable accuracy has been demonstrated for lymph node detection by EAUS [3]. However, data on the accuracy of EAUSs outside large tertiary medical centers have demonstrated lower correlation rates between EAUSs and surgical staging [4]. This finding is probably related to the higher levels of experience among examiners and the higher number of examinations performed within large medical centers [5, 6]. Therefore, an operator-supporting system using artificial intelligence to detect rectal cancer may increase the accuracy of EAUSs performed by less experienced professionals.

Advances in computerized image processing support the development of deep learning (DL) models for image classification. DL techniques have shown great promise in medical image analysis. In the last few years, the use of DL in image segmentation, recognition, and registration has accelerated, and DL algorithms have been demonstrated to learn which feature space is most appropriate for the task at hand [7]. Moreover, DL models are particularly useful in diagnosing and treating colorectal cancer [8]. A convolutional neural network (CNN) architecture is a DL model with an artificial neural network that uses images as input. The use of CNN architecture is rapidly expanding in the field of medicine and has been used in the field of rectal cancers for automatic T-staging of rectal cancers in MRI images [9, 10].

A particularly promising direction in using DL models for medical imaging is self-supervised learning (SSL) [11]. SSL is a type of machine learning that trains a model on unlabeled data. One SSL method that is especially relevant to medical imaging is contrastive learning. The goal of contrastive learning is to learn a feature representation space where similar patches of an image are brought closer together while dissimilar samples are pushed farther apart. Recent results show the particular applicability of this method to ultrasound classification tasks [12].

While previous studies on the DL-assisted diagnosis of colorectal cancer have concentrated on MRI and CT imaging [9], the purpose of this study was to develop and validate DL models that can distinguish rectal cancer from standard rectal EAUS images since ultrasound imaging is a significantly more affordable technique than MRI and CT. The acceptable accuracy of EAUSs may significantly improve the outreach of medical services.

## Methods

A prospective EAUS image database was reviewed to identify examinations performed for primary stage rectal cancer. We included all examinations performed between February 1, 2021, and December 31, 2021. A total of 40 patients were included in this study, and 294 two-dimensional (2D) images were extracted for analysis (161 abnormal, 133 normal) (Table 1). The study was approved by the Sheba Medical Center ethics committee.

**Table 1:** Cohort and disease characteristics

Male	24 (60%)
Female	16 (40%)
Age (Ys)	$61\pm 22$
Tumor invasiveness (T stage)	
T0	11 (27.5%)
T1	13 (32.5%)
T2	4 (10%)
T3	10 (25%)
T4	2 (5%)

### Endoanal ultrasound

**Fig. 1:**
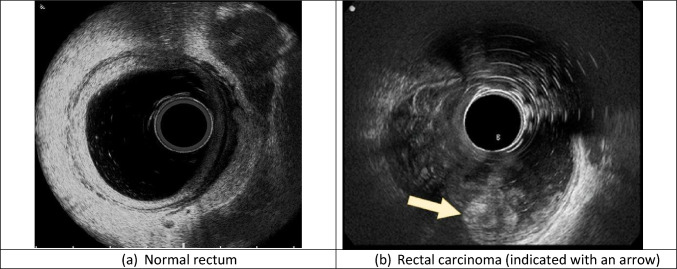
Endoanal ultrasound images of a water-filled rectum

All examinations were performed using a single ultrasound machine (BK 300, Peabody, USA) by two experienced examiners ( > 1000 examinations each) using the same technique. Examinees were instructed to perform two cleansing enemas, one at 2 h before the examination and one at 1 h prior. The rectum was inflated by inserting 250–300 cc of water before inserting the rectal us probe. Images were taken using an automatized 360° radial transrectal transducer with a frequency range of 3–20 MHz (3D 20R3) and were stored on the ultrasound machine (Fig. 1). The ultrasound imaging parameters, such as gain, frequency, and gain compensation, were left to the examiner’s discretion.

### General approach to artificial intelligence

This paragraph explains the basics of the scientific computing performed in this study. A detailed explanation will follow in the next paragraphs.

After the extraction of ultrasound images, all images were categorized as normal (no cancer) or abnormal (cancer) by an experienced sonographer. This step was followed by anonymizing the images by removing any data not related to the images. Image quality was then improved automatically by reprocessing the images to delineate the area of interest within the image. Because we had a limited number of images, we augmented the training dataset by randomly resizing, cropping, and rotating the images. To evaluate the performance of a Convolutional Neural Network (CNN) classifier, a tenfold cross-validation technique was utilized. This technique divided all the images into 10 groups, where each group contained an approximately equal number of images. For every iteration, a single group was used as the test set, while the remaining 9 groups were used for training and validation. This process was repeated 10 times. We used different types of CNN architectures and looked for the one with the best sensitivity for the detection of abnormal images.

### Image extraction and cropping

For each patient, all of the images of the rectum were extracted, including normal and abnormal images. Images were reviewed by a single experienced ultrasonographer (DC) and categorized into two groups based on the positive or negative visualization of the tumor in the image. All patient data was anonymized by cropping the metadata areas to avoid ethical issues. The image resolution of the final output was 797×657 pixels.

### Image preprocessing

Various preprocessing operations were applied to enhance each image for further analysis. First, a mask was created to remove colored marks from the image. The grayscale image was then masked to remove specific pixel values and colored marks, and inpainting was performed on the masked image to fill in missing values. The resulting image was then thresholded (using the value 8 as the threshold) to create a binary image. Next, separated objects were labeled, and the largest bounding box was determined to crop the image.

Further processing involved removing small spots and finding a solid circle, which was masked out from the cropped image. This cropped and processed grayscale image served as the first stage of the preprocessed image. The next step aims to enhance the visual contrast of the images by amplifying the difference between bright and dark areas. To do so, a combination of top hat and bottom hat morphological operations was used. The goal of the top hat operation is to detect bright regions of an image that appear on a darker background, whereas the bottom hat has the opposite goal. Adding the result of the top hat to the original image enhances the bright regions, and subtracting the result of the bottom hat enhances the dark regions. Combining these two operations (therefore making the bright regions brighter and the dark regions darker) increases the contrast in the image. The size of the structuring element used for top hat and bottom hat operations is selected iteratively using the contrast improvement ratio (CIR) measure, which compares the contrast of the original image to the contrast of the final image [13, 14]. The iterative process continues until convergence, meaning that an optimal contrast improvement is achieved. The resulting enhanced grayscale image was then used for classification purposes.

### DL models

One main drawback of CNN architecture is the significant number of labeled images required during the learning process. The general approach to overcome this limitation during classification tasks in medical imaging is to combine transfer learning with fine-tuning [15]. Therefore, our proposed method used state-of-the-art (SOTA) models that were pre-trained to provide feature extraction and further fine-tuned with a relatively small ultrasound image dataset.

### CNN Models

The CNN network cannot be directly trained due to overfitting caused by the limited size of the available database. To enhance performance, transfer learning from a pre-trained CNN was used, followed by fine-tuning. The DL models evaluated in this study are based on the recent SOTA (State of the art) CNNs architectures: Xception [16], InceptionV3 [17], EfficientNet [18], NasNetLarge [19], InceptionResNetV2 [20] and ConvNeXt [21]. The weights of publicly available models of these CNNs were pre-trained on ImageNet. The images in the database were resized based on the input dimensions of the pre-trained CNN.

**Fig. 2:**
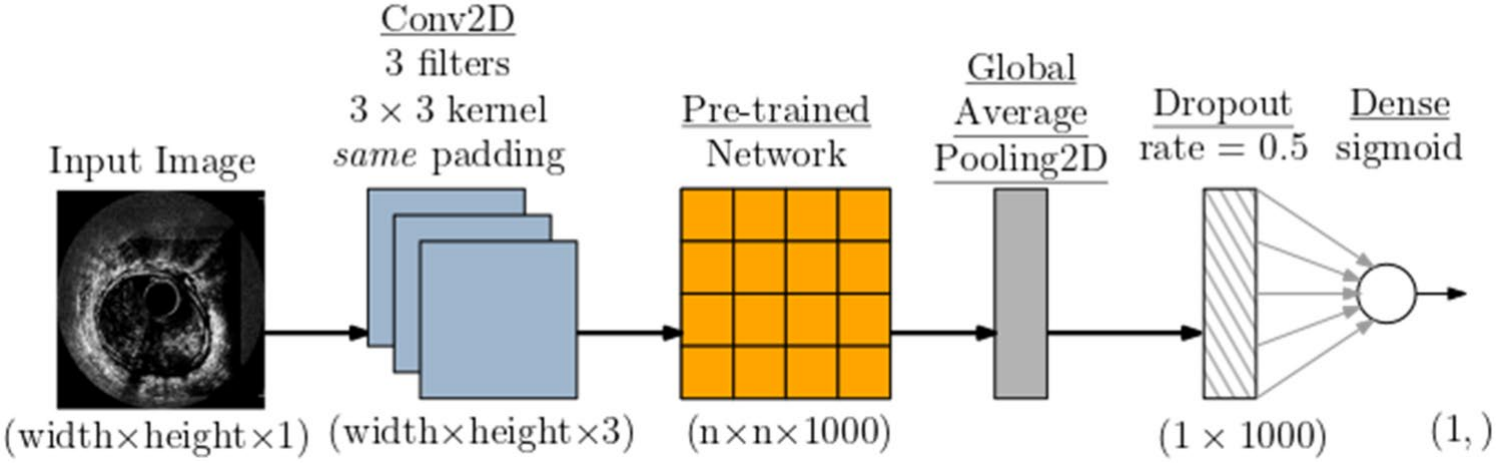
CNN ( convolutional neural network) model architecture

One of the main differences between the common transfer learning procedure and our particular methodology is that CNNs are pre-trained on RGB images, while our images are grayscale. In order to facilitate matching between input grayscale images and pre-trained Convolutional Neural Networks (CNNs), we employed a specialized input layer positioned at the bottom of the pre-trained CNN. This input layer was realized through a Convolutional 2D (Conv2D) layer, consisting of three filters with dimensions of 3 × 3, accompanied by same-size padding [22]. This approach enabled seamless integration of grayscale images with CNN models designed for RGB inputs without the need for any additional modifications to the pre-trained model. The top layer of the pre-trained network was replaced with the global average pooling layer. To reduce overfitting, an aggressive dropout rate of 0.5 was applied. We also tested a lower dropout rate, which resulted in significant overfitting of a training set in the preliminary experiments. Finally, the dropout output was connected to a fully connected layer with a sigmoid activation function. Figure 2 presents the resulting network architecture.

In order to facilitate matching between input grayscale images and pre-trained Convolutional Neural Networks (CNNs), we employed a specialized input layer positioned at the bottom of the pre-trained CNN. This input layer was realized through a Convolutional 2D (Conv2D) layer, consisting of three filters with dimensions of 3 × 3, accompanied by same-size padding. Additionally, the Conv2D layer was equipped with a learnable grayscale-to-RGB conversion feature. This approach enabled seamless integration of grayscale images with CNN models designed for RGB inputs, without the need for any additional modifications to the pre-trained model.

The first training stage was transfer learning, in which layers of pre-trained CNN were frozen, and only the added layers were trained. The second stage was fine-tuning with end-to-end training and a significantly decreased learning rate. An early stopping strategy was applied for both stages based on the classification performance on the validation dataset. Adam optimizer was used for training, in which the original images were resampled to the height and width of the pre-trained network. To enhance the learning progress, the training dataset (but not the test dataset) was randomly augmented with small brightness changes, zoom changes, and an arbitrary rotation angle.

A tenfold cross-validation was used to train the CNN and test classification performance. Specifically, in each round of cross-validation, 10% was used for testing, 15% for validation, and 75% for training. To ensure optimal performance, the early stopping strategy was implemented by monitoring the classification accuracy of the validation dataset. If there was no improvement in accuracy for four consecutive epochs, the training process was stopped, and model weights with the best performance were restored.

Beyond the standard CNN models, this study also used an ultrasound semi-supervised contrastive learning model [13]. The evaluation results are based on the publicly available pre-trained ResNet18 model and the supplementary code. The layers of pre-trained CNN were frozen, except the last three layers. Only these layers and the added fully-connected layer were trained. The training dataset was randomly augmented with resize, crop, and rotate operations, and the test dataset was not augmented. The model was evaluated with tenfold cross-validation, with 10% for testing, 15% for validation, and 75% for training. Significantly, the original code [13] used the same images for validation and testing, but this approach is inappropriate for small databases, such as the one discussed in this paper.

### Development environment

The development environment used to train and test the models was a PC running the Windows operating system on an Intel Core i7-10,700, 2.90-GHz processor CPU, with 64 GB of RAM and an Nvidia RTX A4000 16 GB GPU. The Python 3.9 programming language is used in combination with extension packages, including Tensorflow 2.9, numpy 1.25, scipy 1.3, and confidence interval.

## Results

After the model generated an output for each round of cross-validation, all outputs were accumulated into a probabilistic prediction vector for all the images. Since the outputs appear as a probability, this was converted into a binary decision by comparing it with a classification threshold of 0.5.

Among the evaluated models, the best results were for the EfficientNet model [18] of two different sizes, namely EfficientNetV2M and EfficientNetV2L. The achieved sensitivity for detecting a rectal tumor using the machine learning model was about 0.78, with a specificity of about 0.78 and an accuracy of 0.78. Table 2 displays the corresponding confusion matrices of these models.

**Table 2:** Confusion matrices of the EfficientNetV2L and EfficientNetV2M models that compare actual and predicted values

(a) EfficientNetV2L			
		Predicted	
		Yes	No
Actual	Yes	127	34
	No	28	105

(b) EfficientNetV2M			
		Predicted	
		Yes	No
Actual	Yes	125	36
	No	29	104

The classification performance of all the evaluated models is summarized in Table 3. The confidence intervals were evaluated with the confidence interval package [23]. The contrastive learning model produced slightly lower results than the best CNN model.

**Table 3:** Classification performance for different models

Model	AUC	Accuracy	Sensitivity	Specificity	Precision
EfficientNetV2M	0.853 (0.809, 0.897)	0.779 (0.728, 0.823)	0.782 (0.704, 0.844)	0.776 (0.706, 0.834)	0.743 (0.665, 0.808)
EfficientNetV2L3	0.848 (0.802, 0.893)	0.789 (0.739, 0.832)	0.789 (0.713, 0.85)	0.789 (0.719, 0.845)	0.755 (0.678, 0.819)
EfficientNetV2S	0.74 (0.683, 0.797)	0.69 (0.635, 0.741)	0.729 (0.648, 0.798)	0.658 (0.582, 0.727)	0.638 (0.559, 0.71)
Contrastive learning	0.827 (0.781, 0.874)	0.735 (0.681, 0.782)	0.662 (0.578, 0.737)	0.795 (0.726, 0.85)	0.727 (0.642, 0.799)
InceptionResNetV2	0.798 (0.747, 0.849)	0.724 (0.671, 0.772)	0.699 (0.617, 0.771)	0.745 (0.673, 0.806)	0.694 (0.612, 0.766)
Xception	0.796 (0.744, 0.847)	0.718 (0.664, 0.766)	0.662 (0.578, 0.737)	0.764 (0.693, 0.823)	0.698 (0.613, 0.772)
ConvNeXtLarge	0.791 (0.739, 0.843)	0.707 (0.653, 0.757)	0.797 (0.721, 0.857)	0.634 (0.557, 0.704)	0.642 (0.567, 0.712)
NASNetLarge	0.716 (0.656, 0.776)	0.694 (0.639, 0.744)	0.639 (0.555, 0.716)	0.739 (0.666, 0.801)	0.669 (0.584, 0.745)

Each value represents the mean and the resulting 95% confidence bounds. The results approximately reflect the Top-1 and/or Top-5 ranking of pre-trained CNNs’ performance on the ImageNet dataset

**Fig. 3:**
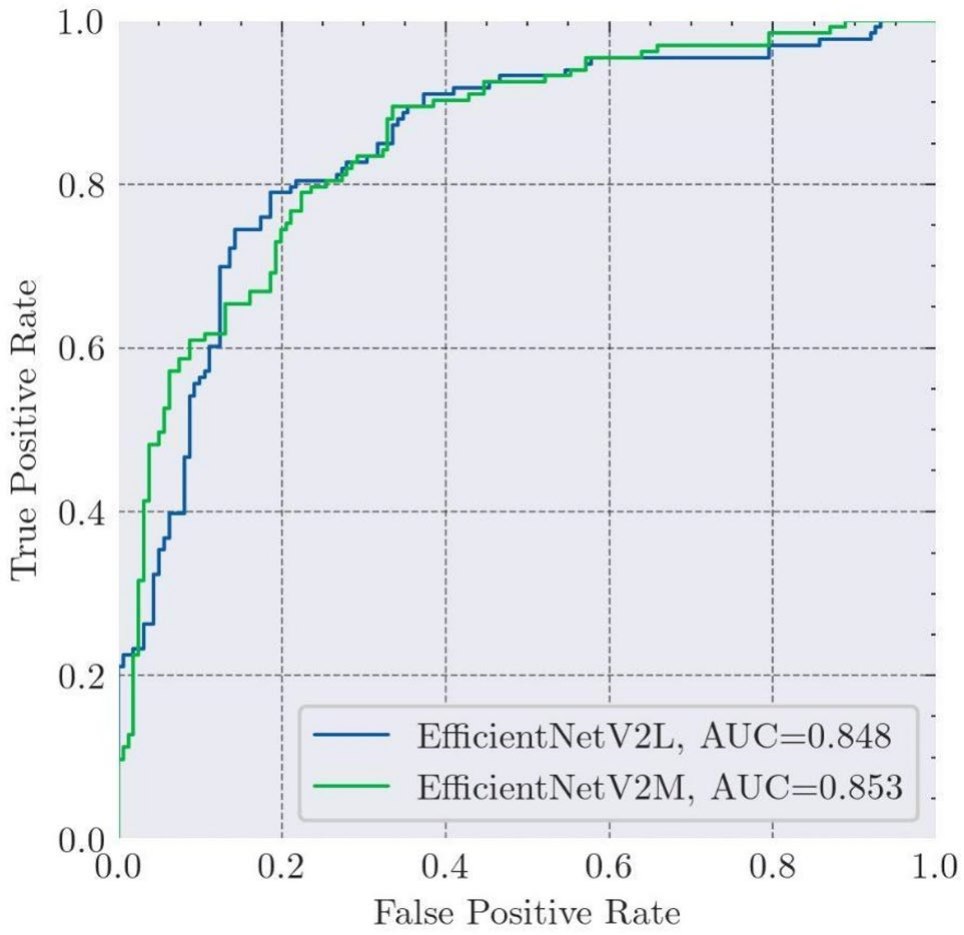
ROC plots of the EfficientNetV2M and EfficientNetV2L models

The receiver operating characteristic (ROC) plot was used for the performance analysis of a model at all classification thresholds. This curve plots the True Positive Rate (TPR) and False Positive Rate (FPR) at different classification thresholds. Figure 3 shows the ROC plot for EfficientNetV2M and EfficientNetV2L models. EfficientNetV2L may have achieved slightly higher accuracy, sensitivity, and specificity, but EfficientNetV2M performs slightly better regarding the area under the curve (AUC).

## Discussion

While CNN has been previously used for various applications in the field of gastroenterology [24–28], our data demonstrates a novel use for a CNN deep learning model: the detection of rectal cancer in EAUS images.

DL models are typically used in gastroenterology to detect and classify pathological processes in the gastrointestinal tract. While applying different DL models to our EAUS image dataset, we achieved sustainable accuracy in detecting rectal cancer and differentiating it from normal rectal images. This has potential clinical implications. First, using an automatized $36{0}^{\circ }$ transducer to perform an EAUS is relatively easy from a technical standpoint, but interpreting the images is demanding and requires expertise. Therefore, applying an automated system that can diagnose rectal cancer as a first stage of assessment of rectal cancer can potentially enable the use of EAUSs by less experienced medical personnel. A positive result, however, would require further assessment by an experienced ultra-sonographer. Another potential use case for this system is as an assistant to decision-making for examiners with limited or no experience with EAUSs. The fact that sustainable accuracy was achieved despite a relatively small image dataset may indicate that developing an AI system for detecting and staging rectal cancer is an achievable goal.

Our study has some limitations, however. Staging of rectal cancer is the main application of EAUSs, but this study only represents the first step in achieving this goal. The use of a small size population and a relatively restricted image dataset limited the ability to teach the model to differentiate between different stages of tumor invasion. Therefore, our group intends to develop a similar AI system for the staging of rectal cancer on a larger image dataset.

Nevertheless, this study demonstrated the feasibility of detecting rectal cancer in EAUS images using DL. Further studies are needed to validate our results during real-life EAUS examinations and to develop an AI module for rectal cancer staging.

## Data Availability

The anonymized data used in this study will be made available by contacting the corresponding author upon reasonable request.

## Abbreviations

EAUS: Endoanal ultrasound
DL: Deep learning
CNN: Convolutional neural network
SSL: Self-supervised learning
MRI: Magnetic resonance imaging
CT: Computed tomography
CIR: Contrast improvement ratio
SOTA: State-of-the-art
ROC: Receiver operating characteristic
TPR: True Positive Rate
FPR: False Positive Rate
AUC: Area under the curve
